# Climate-related migration and population health: social science-oriented dynamic simulation model

**DOI:** 10.1186/s12889-020-10120-w

**Published:** 2021-03-26

**Authors:** Rafael Reuveny

**Affiliations:** School of Public and Environmental Affairs, Indiana University, Bloomington, USA

**Keywords:** Interdisciplinary, System, Mathematical, Numerical, Sensitivity analysis, Validation

## Abstract

**Background:**

Social science models find the ecological impacts of climate change (EICC) contribute to internal migration in developing countries and, less so, international migration. Projections expect massive climate-related migration in this century. Nascent research calls to study health, migration, population, and armed conflict potential together, accounting for EICC and other factors. System science offers a way: develop a dynamic simulation model (DSM). We aim to validate the feasibility and usefulness of a pilot DSM intended to serve as a proof-of-concept and a basis for identifying model extensions to make it less simplified and more realistic.

**Methods:**

Studies have separately examined essential parts. Our DSM integrates their results and computes composites of health problems (HP), health care (HC), non-EICC environmental health problems (EP), and environmental health services (ES) by origin site and by immigrants and natives in a destination site, and conflict risk and intensity per area. The exogenous variables include composites of EICC, sociopolitical, economic, and other factors. We simulate the model for synthetic input values and conduct sensitivity analyses.

**Results:**

The simulation results refer to generic origin and destination sites anywhere on Earth. The effects’ sizes are likely inaccurate from a real-world view, as our input values are synthetic. Their signs and dynamics are plausible, internally consistent, and, like the sizes, respond logically in sensitivity analyses. Climate migration may harm public health in a host area even with perfect HC/ES qualities and full access; and no HP spillovers across groups, conflict, EICC, and EP. Deviations from these conditions may worsen everyone’s health. We consider adaptation options.

**Conclusions:**

This work shows we can start developing DSMs to understand climate migration and public health by examining each case with its own inputs. Validation of our pilot model suggests we can use it as intended. We lay a path to making it more realistic for policy analysis.

**Supplementary information:**

The online version contains supplementary material available at 10.1186/s12889-020-10120-w.

## Background

In 1847–8, a typhus epidemic hit Prussia’s poor Upper Silesia. The government turned to Doctor Virchow for advice, and he traveled to Upper Silesia. “Medicine,” he argued in his report, “is a social science, and politics nothing else but medicine on a grand scale” [1]. His insight is relevant in our era of global climate change. The ecological impacts of climate change (EICC) can harm health directly (e.g., heat-related problems, extreme weather-related trauma) and indirectly (e.g., spreading pathogens, reducing food security, increasing the risk of armed conflict) [2].^1^ Effects may vary by region, but researchers expect they will grow in this century, especially if greenhouse gas emissions continue at current rates. People sidelined socially, politically, or economically are more vulnerable, especially in developing nations (the global South) [3].

EICC may elicit varied reactions. For example, people may do nothing, mitigate emissions, adapt in situ with defenses, or adjust by emigrating. Effective mitigation requires global cooperation. Poor people may be less able to adapt in situ than others and more inclined to relocate, all else the same. We call people migrating due to the combination of EICC and other factors climate migrants. Empirical social science research, we shall see, suggests EICC has contributed to migration in recent decades.

We aim to say something about climate migrants and health. In 2019, there were 272 million (M) authorized international immigrants globally (including 25.9 M refugees, 164 M workers, and 3.5 M asylum-seekers), a new high absolutely and relative to the world population. Most of them moved from the South to developed nations (the Global North), or South-North, as did another 3.9–10 M who were stateless [4]. Another 58 M immigrants were likely unauthorized, many South-North [5], 4 M fled Venezuela [6], and 763 M migrated internally (in-state)—also a new high—most in the South [7].

A recent global survey finds 710 M people wish to migrate, almost all South-North [8]; most may not move. Projections suggest massive migration in this century. The United Nations projects 82 M added South-North net migrants (in-out) by 2050 if the current economic and demographic trends continue [9]. Cities in the South may hold 1.5 billion more people by 2050 [10]; 40% may be net internal migrants [11]. Projections for climate change consider a baseline without adaptation to and mitigation of carbon emissions (business as usual) and global climate tipping points. For example, floods and rising sea levels may add 200 M migrants globally by 2050 [12]. The number of climate migrants by 2100 may be as high as 700 M [13]. By 2050, rising sea levels, declining crop yields, and dwindling safe water may add 143 M net internal migrants in Africa, Latin America, and South Asia [14]. Rising sea levels may add 187 M migrants globally by 2100 [15]. In 2080–99, 236 M people may leave African nations due to heat and rain anomalies [16]. In 2070–99, heat anomalies may add 20 M asylum-seekers [17]. Current data imply these may move mainly within their nations, especially in the South, though many may move abroad.

These figures suggest it is prudent to study population health in the context of climate migrants. Research in this area is emerging and rarely explores climate, migration, and health together [18]. Studies survey health outcomes observed for modern-day immigrants as analogs for future climate migrants. For example, people forced to move by EICC and the associated adaption projects in the future may face infectious diseases, mental health problems, and health risks of EICC, pollution, and waste in coastal cities [2, 19–21]. Planned climate migrants may fare better [22]. Climate migrants may arrive broke and settle in areas facing health risks [10]. Health problems may reduce climate migration and rise on its way [23, 24]. EICC and Climate migrants may raise the risk of conflict in host areas. EICC and conflict may harm health and health care. The need for health care may top the ability to provide it [25, 26]. The topic is complex, involving dynamic interconnections, feedbacks, multiple causal chains, trigger thresholds, integrated effects, and factors like health care, social, economic, political, demographic, environmental, and legal [18, 27, 28].

One way to study complexity is to model it as a mathematical system and simulate it over time. Dynamic simulation models (DSMs) of systems add insight beyond knowing how each of their parts works in statistical models by including interrelationships. They inform how variables change together in response to one another and other factors, unlike statistical models that tell how a variable responds to an input change, holding other inputs constant. DSMs usually aim to project what may happen to variables in the future, unlike statistical models that seek to explain variables based on data, typically one at a time [29]. No one, of course, knows the future. DSM projections depend on their input values (storyline) and, like all models, simplify reality.

Building on Virchow’s idea of health as social, we take a system science approach to population health. Populations are complex systems, and examining their health can benefit from modeling it together with population size, migration, and the potential for conflict over time, taking account of the ecological impacts of climate change (EICC), social determinants of health, and other factors [24, 25, 28, 30, 31]. Demand for such DSMs is rising among a broad range of stakeholders [28, 32].

A survey of theory-based empirical processes in critical domains provides a natural starting point for DSM design. The survey results, presented in the next section, paint a complex interconnected and interdisciplinary picture occasioned by inequalities in health, environmental health, and access to health care and environmental health services for immigrants compared to native hosts, especially for those coming from or migrating internally in developing countries. The associated complexity may explain why social science has not developed a DSM of the type we propose despite those calls and rising demand.

One challenge of modeling dynamic systems is the temptation to explain “too much.” Thinking “too big” may create a model in which everything affects everything else, making it hard to understand anything; it is better to model some things as exogenous [33]. The climate migration-public health picture is complex and hard to model in one fell swoop, even after defining some factors as exogenous. We do not have a magic solution to address this interdisciplinary complexity alone, so we turn to one of the oldest tricks in the “book of modeler”: start simple to get the ball rolling.

We aim to validate the feasibility and usefulness of a basic DSM intended to serve as a basis for identifying extensions to make it more realistic. Our model is a pilot, a computational proof-of-concept illustrating that we can and should do more to develop DSMs of climate migration and population health. It simplifies reality, but it is already rigorous, resulting in many variables and parameters. Our pilot model depicts people migrating each period and joining their brethren in a host area. The origin and destination sites can be in the same country or different countries. An origin has one group of residents, its population, and a host site has two groups, immigrants and native hosts. A group needs health care (HC) depending on its health problems (HP) and environmental health services (ES) depending on the non-EICC environmental health problems (EP) it faces (e.g., pollution, waste, toxic materials). A site may have EICC and conflict. The model computes composites of all intrastate conflict types per site, all HP pc, EP, HC, ES, HC quality, and ES quality types, in turn, per group; population per group; HC and ES capacities per site; and the number of migrants per period by the OD pair and their HP pc on arrival. These variables affect one another and respond to respective exogenous composites of EICC, HC barriers, and social, political, economic, natural, demographic, and geographic factors.

We simulate our pilot DSM for reasonable synthetic values since the literature does not measure its composites as such. The sizes of the obtained effects are thus likely inexact from a real-world viewpoint. However, their directions and dynamic patterns are plausible and, like the sizes, respond logically in sensitivity analyses. Our pilot DSM has conceptual validity since it integrates theory-based empirical mechanisms. It has plausibility, internal, and sensitivity validities since it delivers sensible results that agree with associated theories. It is thus valid enough for its stated intended use. We suggest modeling extensions to increase model realism and lay out a path on how to do that.

A fully validated DSM examining interrelationships between migration, population, public health, and armed conflict under climate change for given input values could suggest conditional answers to relevant questions. For example, how many migrants may come to a host area when certain EICC intensify in their origin site, other things the same? How healthy could they be on arrival? If a certain number arrives over a specified duration, how will the native-host health change? If EICC in their origin harm their health, what may be the health effects in their host area? What may be the effects if host authorities limit their access to HC and ES available for native hosts? Will the host’s HC and ES systems suffice to address the total need as climate migrants arrive? Could armed conflict occur in the host area, and if so, what might be its HP, EP, HC, and ES impacts? Given the projected rise in global migration due to changes in EICC, demographic profiles, and other factors, as well as the current HP, EP, HC, and ES inequalities for internal immigrants in internal immigration hubs such as urban China and India, and international in almost all nations, developing such a DSM is more pertinent now than ever before.

## Methods

Nascent public health research treats health outcomes observed for modern-day migrants as analogs for the future climate migrants. Empirical social science research separately studies essential domains in the context of modern-day migration. Surveying the associated results provides a logical starting point for DSM design. Our survey does not aim to contradict (or support) reported findings. We take them all at face value and merge their causal processes with system science principles in designing our DSM. The idea guiding our DSM design, in other words, is not to arbiter disagreements on the signs, sizes, and significance of observed effects but rather to inform possible impacts of different assumptions on the projected system behavior by setting the input values accordingly.

### Empirical social science research

We start with empirical results on the factors of migration. We then discuss results for the roles of immigration and the ecological impacts of climate change (EICC) in conflict. Findings on exposures to EICC and other environmental health problems (EP), health care (HC) use, HC access, HC quality, and health problems (HP) in the context of immigrants provide the next anchors for our discussion. Finally, we examine the social determinants of health and the impacts of conflict on HP and EP. The overall research is too large to cover fully here. We summarize and cite examples.

#### Migration

A large empirical modeling literature explains migration. Results for permanent authorized international immigrants (PIMs) [34], authorized temporary international immigrants (TIMs) [35], unauthorized international immigrants [36], and internal-migrants [37] are quite similar. Migration rises with origin site factors such as poverty, joblessness, and conflict; opposite forces in a destination site; population size in each site; OD-pair proximity, amity, ease of entry, diaspora, and shared language; and the difficulty of moving elsewhere. Refugees and asylum-seekers migrate primarily due to conflict and repression in their origin site, but otherwise, follow suit [38].

New models find international migration rises with environmental changes of the type associated with EICC in sites of origin (rain decline [39], more frequent and intense extreme weather events [40], rain and heat anomalies [41], storms [42]), controlling for those other factors. Current data, as noted, say more people migrate internally than abroad, especially in the South. Results show more EP [43], extreme weather events [44], droughts [45], heat, and storms [46] in origins raise internal emigration. Still, there are more possibilities. Sufficiently large EICC-related impacts and financial losses can hinder the ability to migrate abroad, especially from developing nations [47].

The overall impact of conflict on migration also reflects competing forces. Studies generally find that conflict events in origins site increase emigration, as noted. However, logic and some findings suggest that severe enough conflict events can reduce the abilities to migrate and to cope in situ with EICC; the latter effect, in turn, can raise emigration from impacted regions or, if it is large enough, reduce the outflow [48].

Historically, epidemics contributed to migration from affected areas. The Black Death, e.g., led to migration in Europe and the Middle East. Newer cases include the migrations linked to the 1800s cholera and smallpox in Holland [49]), 2008–9 cholera in Zimbabwe [50], 2009 influenza in Mexico, 1994 plague in India, and 2002–3 SARS in China [51]. A recent statistical model finds epidemics promote emigration worldwide, controlling for other factors [52]. Research on the role of modern-day outbreaks in migration is emerging. Logically, there are more possibilities. Epidemics may have little effect on the current migration as they usually have remedies, and governments curtail mobility in response. They may also reduce the ability to move if they have no readily available cures or vaccines.

#### Armed conflict

Models find the risk of conflict rises with population and the prior conflict risk and level [53] [54], controlling for other factors (e.g., economic, political). New models find areas with more refugees and asylum-seekers are at higher risks of terror attacks [55] and civil wars [56], and with more foreign immigrants terror attacks [57] and interstate conflict [58]. Internal immigration contributes to civil strife [59, 60]. The International Panel on Climate Change finds EICC amplify conflict risk factors such as economic decline, ethnic tension, poverty, inequality, and grievance, especially in the South [3]. Newer models find more extreme weather events [61], temperature/rainfall anomalies [62], and droughts [60], and higher peak temperatures [63] raise civil conflict risk, controlling for other factors. States with less rainfall and stronger extreme weather events [64], more extreme weather events [65], and more variable rainfall [66] are at higher risk of interstate conflict.

Epidemics have long fueled armed collective violence. Examples include the Black Death peaking in the Middle East and Europe in the fourteenth century and recurring well into the 18th, cholera in Europe in the nineteenth century, plague in India (1896–1914), Spanish Flu in the United States (1918–20), HIV/AIDS in Zimbabwe in the 1990s and 2000s [67, 68], and Covid− 19 in Columbia, Yemen, Africa, and Ukraine as of 2020 [69]. Nascent models find epidemics fueled wars in China from 1470 to 1911, taking account of other factors [70]. Results show states with higher rates of vector-borne parasitic diseases [71] and more HIV/AIDS [72], and areas with more Ebola in West Africa [73] and Covid− 19 in Burkina Faso, Libya, Mozambique [74] and India [75] have more conflict. In Africa, areas with high and low malaria rates are at a smaller risk of conflict than areas with moderate rates [76].

#### Ecological impacts of climate change (EICC) and non-EICC environmental health problems (EP)

Emerging research finds that areas with more South-North immigrants are at higher flood risk and less flood defense, response, and aid, and slower recovery (Florida [77], Texas [78]). In contrast, high-status coastal areas attract the affluent, where the costs of risk mitigation are partly carried by the broader public [79]. Residential segregation, affordability, and real estate industry practices contribute to these patterns [78]. Other recent studies find areas with more South-North immigrants face more EP harmful for health, controlling for factors like income, production, and population. Studies usually ascribe this pattern to anti-immigrant bias in EP regulation, and immigrant weakness in not-in-my-backyard lobbying, and inability to find cleaner jobs and afford cleaner areas. Examples include exposure to pesticides [80], waste burners [81], industrial/auto emissions [82], fine-particle air pollution [83], and industrial toxins [84]. In China, rural-urban immigrants are more exposed than city natives to landfills, noise, emissions [85], and ground-ozone [86], and in Mexico to pesticides [87].

#### Health care (HC) access

Internal immigrants access HC like locals except in nations linking it to a registered residence and making it hard to change status (e.g., China [88]). Migration abroad can improve HC access compared to that in the origin site. However, the rights of foreign immigrants for HC vary by country and are usually complex matters of law that go by immigrant features like type and age-group. We summarize some conditions.

In the United States, e.g., arriving permanent authorized international immigrants (PIMs) and temporary authorized international immigrants (TIMs) access like citizens but wait five years for funded care if low income [89]. In Canada, new PIMs buy HC or private insurance for several months, then get access; TIMs get partial access [90]. In Australia, PIMs, and some TIMs (e.g., applicants for permanent stay, workers) get access [91]. In the European Union, PIMs and TIMs get full access in 10 states (e.g., Germany, France) and conditional (e.g., work permit) in 22 (e.g., Spain, Britain) [92]. Malaysia requires buying limited private insurance [93]. South Africa [94] and Thailand [93] give access, and Kenya charges more than nationals [95]. In Turkey, new PIMs buy HC or private insurance for eight months and then get access; TIMs get only emergency care (EC) [96] as do PIMs and TIMs in Russia [97].

Among signers of refugee treaties, the United States gives refugees funded HC up to eight months and then access [98], Australia gives access and free initial HC [99], and Canada partial up to a year and then full [100]. The European Union [101], Turkey [96], Kenya [95], and Russia [102] give access and South Africa basic HC [94]. Among non-signers, Malaysia bills more [103], and Thailand treats refugees like unauthorized international immigrants [104]. The United States, Britain [105], and Australia [106] may detain Asylum-seekers with limited HC. Those allowed to live in the community get access to HC in the United States [107] and partial access in Canada [100]. In the European Union, seven states give access (e.g., France), seven conditional access (e.g., stay in centers, Greece), seven partial (Sweden), ten partial and contingent (Portugal), and two only emergency care (Germany) [81]. Thailand and Malaysia give partial access [108]; South Africa rudimentary care [94]; Turkey access to asylum-seekers from the European Union, Turkey, and Syria, and partial access to others [109]; and Kenya bills more [95].

In Canada [90] and the United States [110], Unauthorized international immigrants get only emergency care with pay and, maybe, funded emergency care and open center HC if low income. Australia gives limited access [111]. Five European Union states give conditional access (France), three give partial (Italy), four give partial and conditional (Spain), eight give only emergency care (Germany). Seven give ad hoc emergency care (Britain), and six only paid emergency care (Norway) [92]. South Africa offers basic HC [94], and Russia limited access with pay [97]. Malaysia provides partial access with payment, Thailand partial and costlier access in the area of residence [93], Turkey only emergency care with pay [101], and Kenya only HC in open centers [95].

South-North immigrants may face more barriers to access, e.g., language, cost, limited info, red tape, and delay (United States [112], Canada [113], European Union [114], Scandinavia [115], Australia [116]). South-South foreign migrants may also face long travel for HC, xenophobia, and claims they drain HC and bring disease (Kenya [95], Africa [117], Southeast Asia [108], South Africa [94, 118]). Provider attitudes may stand in the way [119]. Canadian providers may be aloof toward South-North immigrants, use racial slurs [120], and see them as finicky and substance abusers [121]. Portuguese may see them as violent [122]; Belgian wanton [123]; Norwegian different, Dutch difficult, and Swedish rude [124]. HC staff may also tell South-North unauthorized immigrants to pay upfront, reject them as regular patients, deny them care, and report them to the authorities (the North [125], European Union [126]). Providers in the United States may see them as spreading crime and drugs and raising cost and job losses for natives [127], and French as faking illness to be allowed to stay [128].

#### Health care (HC) use and provided quality

Models compare HC use for natives and immigrants, usually South-North, using surveys or records in one country, controlling for, e.g., health, income, insurance, and age. Results show South-North immigrants in the United States generally use less HC than natives (emergency care [129], mental [130], primary care [131]). They tend to overuse emergency care for regular care and underuse other HC in countries that offer free and low-cost emergency care (Australia [132], Canada [133], France [134], European Union [135]). South-south foreign immigrants (South Africa [136], Russia [97], Malaysia [137]) and rural-urban internal immigrants follow suits (China [138], India [139]). Refugees and asylum-seekers tend to use more HC than natives (United States [112], Canada [140], Britain [141], Germany [142], European Union [143], Thailand, Kenya [144]). New models study the unmet need for HC (non-use when needed), controlling for those factors. Results show South-North immigrants are at higher odds of unmet need than natives (United States [145], Norway [146], Italy [147], Holland [148]).

Studies defined HC quality as the extent that the delivered HC is safe, scientific, warranted, patient-centered, efficient, and timely [149]. Statistical models of the type employed for HC use find that South-North immigrants in the United States are at higher risk of lower HC quality in general non-patient centered HC [150], and fewer offered cancer therapies [151]. Elsewhere, they are at higher risk of generic or flawed care in Spain [152], subpar maternal care in France [153] and other European Union states [154], and inadequate psychiatric care in Sweden and Canada [124]. Unauthorized international immigrants are at higher risk of being shifted to other providers in the European Union [155] and of not being slated for follow-ups in Denmark and Belgium [119]. Refugees and asylum-seekers are at higher risk for subpar psychiatric care in Switzerland [156] and suboptimal maternal care in Iran [157]. South-North immigrants are at higher risk for needless hospitalization (preventable with primary care) in Britain [158], United States and New Zealand [159], and Singapore [160], and extended hospital stay (compared to a mean by diagnosis and treatment) and unplanned hospital readmission (within 30 days after discharge) in Holland [161], controlling for those factors.

#### Health problems (HP)

European colonizers introduced new infectious diseases into native societies. The results were often catastrophic. Many studies examine immigration as a “health threat” and stress screening and isolation [162]. Current immigrants from the South are sometimes falsely blamed for spreading infectious diseases, though some South-North immigrants, especially refugees, asylum-seekers, come from places with disrupted HC [163]. Today, infectious diseases usually have remedies, and their effects in the North are relatively small [164]. Impacts are often more significant in the South, where HC systems are weaker [117].

Models examine health using surveys and records in one nation. Many find that authorized permanent and temporary South-North immigrants are healthier than natives upon arrival, controlling for HC and those factors. Examples include better blood pressure and birth outcomes in the United States [165]; health status in the United States, Australia, Britain, Canada [166], and the European Union [167]; mortality in Canada [168]; and mental health in Britain [169]). Reasons suggest ailing people may not migrate, ailing immigrants may return, developed countries screen South-North immigrant health for entry, and immigrants support each other [170, 171]. The effect wanes over time in the host area due to immigrant alienation, poverty, assimilation stress, and unhealthy diet [171].

Others find similar or higher risk for these immigrants (chronic US, Australia, Canada [166]; perinatal US, European Union [170]; mental US, Australia, Canada, European Union [172]; ischemia, stroke European Union [173]; typical HP Sweden [174]; health status Switzerland [175]). Often holding unsafe, dirty, and manual jobs shunned by natives, they are at higher risk for injury, skin, respiratory, perinatal, mental, and musculoskeletal HP [176]. Children and elders often have HP (Canada [177], North [178]). Refugees and asylum-seekers face conflict and usually reside in crude camps on their way. They are at higher risk of MRSA (Holland [179]), tuberculosis (Germany [180]), oral HP (Australia, US, Canada, European Union [181]), perinatal and mental HP (Australia [182, 183]), mental HP (United States [184]), and mental and digestive HP, diabetes, and orthopedic HP (Britain [185]). Detention of asylum-seekers and unauthorized international immigrants harms their mental health (Australia [186], Britain, Canada [187], United States [188]).

For the South, some models find rural-urban internal migrants in China are healthier than city dwellers (e.g., health status [189]); others do not (maternal/mental HP, infectious diseases [190], overall [191], child mental HP [192]). In India, such migrants are at higher risk for mental HP, infectious diseases, mother underweight, and child anemia and stunted growth [139]. South-South foreign immigrants are at higher risk of child mortality in South Africa [193] and Kenya [194], and Malaria, HIV/AIDS, and tuberculosis in South Africa [117]. Refugees, asylum-seekers, and unauthorized foreign immigrants are at higher risk for mental HP in South Africa [194] and Nigeria [195]; mental HP [196] and infectious diseases [197] in Bangladesh; Hepatitis B in Iraq [198]; and physical trauma in Turkey [199].

#### Social determinants of health

Growing social science modeling research examines the impacts of social factors on group health measures such as life expectancy and specific HP’s risk, usually for state or substate units. Results reveal the positive effects of income inequality on heart attack risk at the United States state level [200]; social spending on life expectancy in Canadian provinces [201]; medical technology on life expectancy in developed nations [202]; democracy and health and education expenditures on life expectancy in Asian states [203]; and air pollution on mortality in Chinese counties [204] and Californian areas [205]. Non-whiteness promotes premature birth in the United States [206], discrimination harms mental and cardiovascular health, and low socioeconomic status harms health [207]. Few models study migration. In the European Union, South-North immigrants in states with policies that exclude immigrants and foster assimilation are less healthy than those without such policies [208]. Pro health spending and equity policies in the European Union help natives more than immigrants (due to HC barriers) [209].

#### Conflict impacts on HP, HC, and EP

New studies find conflict harms health directly (e.g., injury, mental trauma, worsening existing HP) and indirectly (e.g., causing EP, damaging HC, reducing food security). Children, women, the elderly, and relegated groups are at higher risk [210, 211]. Conflict disperses infectious diseases by moving carriers, crowding refugees, impeding eradication, and lowering immunity (e.g., France-Italy wars (the 1500s) syphilis; Napoleonic wars typhus; Crimean war (1854–6) dysentery; France-Prussia war (1870–1) smallpox; World War I influenza; Afghanistan war tuberculosis [67]). In the 2010s, it spread cholera in Africa and Yemen, yellow fever in Angola, infectious diseases in Syria, and polio in Afghanistan, Pakistan, Iraq, and Somalia [212, 213]. Nascent models find conflict raises the risk of leishmaniasis in the South [214], infant mortality and stunted child growth in Africa [215], HIV/AIDS Africa [216], and Ebola in Congo DR [217], controlling for other factors. Immigrants from conflict areas are at higher risk of mental HP than residents (Canada [218]). Conflict disrupts HC in Africa, and lowers the odds of birth in medical facilities [219], creates EP [220], and disrupts ES [221] (e.g., Iraq, Vietnam, the South [222]). Nascent models find similar results for EP [221, 223].

### Pilot model

We join these results with system modeling. Time (t) moves from 0 in periods. The model computes stocks (totals by t), flows (stock changes per period), and auxiliary variables. Its inputs are parameters, time series (scenarios), initial stocks, initial lagged variables, and controllers (tell whether to compute some variables or set them to given values, for flexibility). At the start of a simulation, the model assigns selected values to all its inputs. It then computes by period for the desired simulation duration.

The model simplifies reality in line with our stated plan to do so. It depicts a system with one origin and one destination and no inter-site conflict. The migration process ends within a period. The period size is one in unnamed units, and the variables are composites. The origin site has one population, and the destination has two, natives and immigrants, which do not intermix. We revisit these simplifying assumptions and evaluate them later.

The algorithm is general. The migration can be internal or international. Groups have individual stocks and flows of HP pc, EP, population, and variables for HC/ES needs and provisions, qualities of provided HC/ES when needed, and barriers (legal and otherwise) to access HC/ES. Sites have individual variables for HC/ES capacities (highest service volumes due to, e.g., existing HC providers/hospitals for HC, and waste removal/treatment facilities for ES). HP pc rising above a threshold raises HC’s need, and above a higher level, death. EP follows suits with levels for needing ES and increasing HP pc. A unit of provided HC/ES with a perfect quality reduces HP pc/EP by one. The exogenous variables (inputs) include EICC per site, HC/ES capacities without EICC and conflict per site, HC/ES qualities without these forces per group, HC/ES barriers per group, and integrated effects of non-EICC (TNE) factors such as social, economic, and political on computed variables.

One may present DSMs in several ways (e.g., equations, a diagram, in the main text, in an appendix). We show the math for sharpness, though, in truth, there is no perfect or standard way to show DSMs. Following the math may require careful reading as our DSM includes many equations, variables, and parameters. We hope that our approach of defining the variables and parameters when introduced and in a list available online in Additional file 1: Appendix under the title Supplementary Information at the end of the paper and devising informative notation rules would help.

Variable names use the form X_Y_Z_T_. X is o for origin, d destination, od pair, dn destination natives, and di destination immigrants. Y is a label; it ends with pc for per capita. Z is s for stocks, f for flows, p for parameters, a for auxiliaries, x for exogenous variables, and tx for TNE effects. T is t in this period, t + 1 the next, t − 1 prior. Parameters exclude T and names shared by groups/sites X. Flows, rates, TNE effects, and some random draws can take any value. Other variables vary in ranges or are ≥0. We use five functions. R(x, p) depends on input x ≥ 0 and parameter p: R = 0 for 0 ≤ x ≤ p and rises with x for x > p. RF(x, p_1_, p_m_, p_2_, f_m_) depends on x ≥ 0 and parameters 0 ≤ p_1_ ≤ p_m_ ≤ p_2_ and f_m_ ≥ 0: RF = 0 for 0 ≤ x ≤ p1; rises with x for p1 ≤ x < p_m_; = f_m_ for x = p_m_; falls above 0 as x rises from p_m_ to p_2_; = 0 for x = p_2_; and falls below 0 as x rises above p_2_. R/RF give zero if all their parameters are zero. We name them serially (their values differ by equation/input). MAX(x, y)**/**MIN(x, y) give the largest/smallest among x and y. RD (pd, ps) randomly draws a number from probability distribution pd. with a parameter set ps.

We present the equations for time t in the order of computation. To simplify, we do not show tests of controllers (if _ c = 1, x = scenario; else, compute x), zero population (if pop _ s = 0, x pc = 0), and range (if x > 1, x = 1), and parameters of the R/RF functions, but they are understood.

#### Conflict

The model compares a computed conflict risk (likelihood) to a threshold randomly drawn from a probability distribution defined on the range 0 to 1. If the risk tops that threshold, the model computes conflict intensity; else, it sets the conflict intensity to 0 (none). We use a uniform distribution, assuming a site has a neutral conflict proneness (any threshold is equally likely to be chosen). For a conflict-prone site, one would use a right-skewed distribution (tail on the right), making it easier for the computed risk to top the threshold, and vice versa for peace-prone.

The risk of conflict in the origin site (o_cr_a) rises when the site’s population stock (o_pop_s) and the prior conflict risk (o _ cr _ a_t − 1_) top respective thresholds in R functions. The impacts of the EICC and previous conflict intensity follow RF functions (first increase and then decline due to, e.g., damage to arms and the ability to fight). The effect of HP pc also follows an RF function (rises and then falls as people become less able to fight due to, e.g., morbidity and, for infectious diseases like cholera, high exposure promoting immunity and thus reducing the pro-conflict effect [76]). The TNE effect follows scenario o_cr_tx. Not shown, the model keeps the computed conflict risk between 0 and 1 (as it is a probability).

1. **o**_**cr**_**a**_**t**_ **= R**_**1**_(**o**_**pop**_**s**_**t**_) **+ R**_**2**_(**o**_**cr**_**a**_**t** ‐ **1**_) **+ RF**_**1**_(**o**_**eicc**_**x**_**t**_) **+ RF**_**2**_(**o**_**ci**_**a**_**t** ‐ **1**_) **+ RF**_**3**_(**o**_**hppc**_**s**_**t**_) **+** **o**_**cr**_**tx**_**t**_

The model randomly draws a risk threshold from a uniform distribution, compares it to o_cr_a, and computes the conflict intensity (o_ci_a), or sets it to zero, accordingly.

2. **if o**_**cr**_**a**_**t**_ ≤ **RD**(**uniform**, **0**, **1**) **: o**_**ci**_**a**_**t**_ **= 0**
3. **if o**_**cr**_**a**_**t**_ > **RD**(**uniform**, **0**, **1**) **: o**_**ci**_**a**_**t**_ **= R**_**3**_(**o**_**pop**_**s**_**t**_) **+ RF**_**4**_(**o**_**eicc**_**x**_**t**_) **+** **RF**_**5**_(**o**_**ci**_**a**_**t** ‐ **1**_) **+ RF**_**6**_(**o**_**hppc**_**s**_**t**_) **+ o**_**ci**_**tx**_**t**_

Conflict risk in the destination site (d_cr_a) depends on the sizes of the immigrant and native populations (di_pop_s, dn_pop_s), their HP pc stocks (di_hppc_s, dn_hppc_s), and site factors. The model compares the computed risk to a random risk threshold and sets the conflict intensity (d_ci_a) accordingly.

4. **d**_**cr**_**a**_**t**_ **= R**_**4**_(**di**_**pop**_**s**_**t**_) **+ R**_**5**_(**dn**_**pop**_**s**_**t**_) **+ R**_**6**_(**d**_**cr**_**a**_**t** ‐ **1**_) **+ RF**_**7**_(**d**_**eicc**_**x**_**t**_) **+ RF**_**8**_(**d**_**ci**_**a**_**t** ‐ **1**_) **+ RF**_**9**_(**di**_**hppc**_**s**_**t**_) **+ RF**_**10**_(**dn**_**hppc**_**s**_**t**_) **+ d**_**cr**_**tx**_**t**_
5. **if d**_**cr**_**a**_**t**_ ≤ **RD**(**uniform**, **0**, **1**) **: d**_**ci**_**a**_**t**_ **= 0**
6. **if d**_**cr**_**a**_**t**_ **> RD**(**uniform**, **0**, **1**) **: d**_**ci**_**a**_**t**_ **= R**_**7**_(**di**_**pop**_**s**_**t**_) **+ R**_**8**_(**dn**_**pop**_**s**_**t**_) **+ RF**_**11**_(**d**_**eicc**_**x**_**t**_) **+ RF**_**12**_(**d**_**ci**_**a**_**t** ‐ **1**_) **+ RF**_**13**_(**di**_**hppc**_**s**_**t**_) **+ RF**_**14**_(**dn**_**hppc**_**s**_**t**_) **+ d**_**ci**_**tx**_**t**_

#### Arrivals & their HP pc:

A sum of three effects gives the number of immigrants from the origin to the destination in time t (od_nm_a). An OD effect tracks TNE scenario od_nm_tx. An origin effect (o_nm_a) depends on TNE scenario o_nm_tx, rises as the population rises above a threshold, and rises as the HP pc stock, EP stocks, conflict, and EICC increase and then falls as these four forces continue to increase above their distinct migration obstacle levels, in turn. A destination’s effect (d_nm_a) depends on TNE scenario d_nm_tx, increases as the immigrant and native populations rise above respective thresholds, and falls as their HP pc stock (di_hppc_s, dn_hppc_s), the EP stocks they face (di_ep_s, dn_ep_s), and EICC and conflict in the destination rise above respective thresholds.

7. **o**_**nm**_**a**_**t**_ **= R**_**9**_(**o**_**pop**_**s**_**t**_) **+ RF**_**15**_(**o**_**hppc**_**s**_**t**_) **+ RF**_**16**_(**o**_**ep**_**s**_**t**_) **+ RF**_**17**_(**o**_**ci**_**a**_**t**_) **+ RF**_**18**_(**o**_**eicc**_**x**_**t**_) **+ o**_**nm**_**tx**_**t**_
8. **d**_**nm**_**a**_**t**_ **= R**_**10**_(**di**_**pop**_**s**_**t**_) **+ R**_**11**_(**dn**_**pop**_**s**_**t**_) − **R**_**12**_(**di**_**hppc**_**s**_**t**_) − **R**_**13**_(**dn**_**hppc**_**s**_**t**_) − **R**_**14**_(**di**_**ep**_**s**_**t**_) − **R**_**15**_(**dn**_**ep**_**s**_**t**_) − **R**_**16**_(**d**_**ci**_**a**_**t**_) − **R**_**17**_(**d**_**eicc**_**x**_**t**_) + **d**_**nm**_**x**_**t**_
9. **od**_**nm**_**a**_**t**_ **= o**_**nm**_**a**_**t**_ **+ d**_**nm**_**a**_**t**_ **+ od**_**nm**_**tx**_**t**_

The HP pc of the immigrants upon arrival to the destination site (od_imhppc_a) reflects their origin’s HP pc stock, and scenario od_nmhppc_x for the OD emigrant to origin HP pc ratio (value = 1 means emigrants are as healthy as origin people are, < 1 healthier, and > 1 less healthy). This scenario captures emigrant self-selection by health (emigrants may be in better shape than others in the origin site, as migration is taxing, or less healthy and seek better HC) and the health change during the migration.

10. **od**_**imhppc**_**a**_**t**_ ***=*** **o**_**hppc**_**s**_**t**_ ***∗*** **od**_**nmhppc**_**x**_**t**_

#### Origin health problems (HP) per capita (pc) flow:

People need HC to the extent their HP pc stock tops threshold hchppc_p: MAX(o _ hppc _ s_t_ − hchppc _ p, 0). HC need pc faces barriers (scenario o_hcb_x), which vary from 0 (none) to 1 (no access). The total needed HC for provision (o_tnhcfp_a) rises with the need and population and falls due to barriers.

11. **o**_**tnhcfp**_**a**_**t**_ =  **MAX** (**o**_**hppc**_**s**_**t**_ − **hchppc**_**p**, **0**) ∗ (**1** − **o**_**hcb**_**x**_**t**_) ∗ **o**_**pop**_**s**_**t**_

HC quality (o_hcq_a) tracks scenario o_hcq_x with entries from 0 (futile) to 1 (perfect) for a case without conflict and EICC. It falls as conflict and EICC rise above respective thresholds for causing damage.

12. **o**_**hcq**_**a**_**t**_ = **o**_**hcq**_**x**_**t**_ − **R**_**18**_(**o**_**ci**_**a**_**t**_) − **R**_**19**_(**o**_**eicc**_**x**_**t**_)

HC capacity follows scenario o_hcc_x for a case without conflict and EICC and declines as conflict and EICC increase above thresholds for causing damage, in turn.

13. **o**_**hcc**_**a**_**t**_ = **o**_**hcc**_**x**_**t**_ − **R**_**20**_(**o**_**ci**_**a**_**t**_) − **R**_**21**_(**o**_**eicc**_**x**_**t**_)

If the total need for provision (TNFP) of HC is below the HC capacity, the model provides the needed HC. Otherwise, it delivers the HC capacity itself. The HP pc flow impact of HC provision (o_phchppc_a) accounts for HC quality.

14. **o**_**phchppc**_**a**_**t**_ =  **MIN** (**o**_**tnhcfp**_**a**_**t**_,  **o**_**hcc**_**a**_**t**_)/**o**_**pop**_**s**_**t**_ ∗ **o**_**hcq**_**a**_**t**_

The origin’s HP pc stock rises if the emigrant group is healthier than a typical origin person, and vice versa. The linked impact on the HP pc flow (o_emhppc_a) is:^2^

15. **o**_**emhppc**_**a**_**t**_ = **od**_**nm**_**a**_**t**_/(**o**_**pop**_**s**_**t**_ − **od**_**nm**_**a**_**t**_) ∗ (**o**_**hppc**_**s**_**t**_ − **od**_**emhppc**_**a**_**t**_)

Health may change by chance. Densities of HP data tend to be large around some value and steadily fall away from it in either direction [224]. Probability distributions are usually closer to normal in healthy people than in sick [225] and may vary by HP type.^3^ Assuming people tend to be healthy, the model randomly draws the health impact of chance from a normal distribution with mean 0 and variance vhppc_p. Models assuming otherwise or disaggregating HP by type or level may use other distributions. Natural wear & tear (wthppc_p) raises HP pc, and self-healing (shhppc_p) lowers it. The EP stock facing the population (o_ep_s), conflict, and EICC raise HP pc as they rise above respective thresholds. A TNE impact on the HP pc flow tracks scenario o_hppc_tx.

The following equation joins the contributions to get the origin’s HP pc flow (o_hppc_f):

16. **o**_**hppc**_**f**_**t**_ = **wthppc**_**p** + **R**_**22**_(**o**_**ep**_**s**)_**t**_ + **R**_**23**_(**o**_**ci**_**a**_**t**_) + **R**_**24**_(**o**_**eicc**_**a**_**t**_) + **o**_**emhppc**_**a**_**t**_ + **RD**(**Normal**, **0**, **vhppc**_**p**) − **shhppc**_**p** − **o**_**phchppc**_**a**_**t**_ + **o**_**hppc**_**tx**_**t**_

#### Origin environmental health problems excluding EICC (EP) flow:

The expression MAX(o _ ep _ s_t_ − esep _ p, 0) gives the population’s ES need (the extent the EP stock it faces tops threshold esep_p). ES need faces barriers (scenario o_esb_x), which vary from 0 (none) to 1 (no access). The total ES need for provision (o_tnesfp_a) is:

17. **o**_**tnesfp**_**a**_**t**_ =  **MAX** (**o**_**ep**_**s**_**t**_ − **esep**_**p**, **0**) ∗ (**1** − **o**_**esb**_**x**_**t**_)

ES quality in the origin (o_esq_a) varies from 0 (futile) to 1 (perfect). It tracks scenario o_hcq_x with entries from 0 to 1 when there is no conflict, and EICC and falls as conflict and EICC rise above respective thresholds for causing damage.

18. **o**_**esq**_**a**_**t**_ = **o**_**esq**_**x**_**t**_ − **R**_**25**_(**o**_**ci**_**a**_**t**_) − **R**_**26**_(**o**_**eicc**_**x**_**t**_)

ES Capacity follows scenario o_esc_x in the absences of conflict and EICC and declines as conflict and EICC rise above respective thresholds for causing damage.

19. **o**_**esc**_**a**_**t**_ = **o**_**esc**_**x**_**t**_ − **R**_**27**_(**o**_**ci**_**a**_**t**_) − **R**_**28**_(**o**_**eicc**_**x**_**t**_)

If the ES capacity suffices, ES provision equals the total need for provision (TNFP) of ES; else, ES provision equals the ES capacity. The EP flow impact of ES provision (o_tpesep_a) is given by:

20. **o**_**tpesep**_**a**_**t**_ =  **MIN** (**o**_**tnesfp**_**a**_**t**_, **o**_**esc**_**a**_**t**_) ∗ **o**_**esq**_**a**_**t**_

An individual creates o_ieppc_p EP per period (e.g., bio waste, other waste, energy use pollution). Emigration cuts total creation. The flow impact is o _ ieppc _ p ∗ (o _ pop _ s_t_ − od _ nm _ a_t_). EP decays (e.g., breaks down, dissipates) at the rate o_decrep_p. The EP impacts of conflict and EICC rise as creation and ES damage offset harm to creators and then falls as the effects reverse. The TNE impact is o_ep_tx.

The following equation adds the impacts to get the EP flow in the origin site (o_ep_f).

21. **o**_**ep**_**f**_**t**_ = **o**_**ieppc**_**p** ∗ (**o**_**pop**_**s**_**t**_ − **od**_**nm**_**a**_**t**_) + **RF**_**19**_(**o**_**ci**_**a**_**t**_) + **RF**_**20**_(**o**_**eicc**_**x**_**t**_) − **o**_**tpesep**_**a**_**t**_ − **o**_**decrep**_**p** ∗ **o**_**ep**_**s**_**t**_ + **o**_**ep**_**tx**

#### Origin population flow:

Parameter o_netbr_p gives the origin population’s natural net birth rate (birth rate minus death rate). The population growth rate depends on a TNE impact (o_popgr_tx) and falls due to emigration, and when the HP pc stock exceeds its death threshold. The HP pc death level tops that for HC need. The next equation gives the population flow (o_pop_f).

22. **o**_**pop**_**f**_**t**_ = **o**_**pop**_**s**_**t**_ ∗ (**o**_**netbr**_**p** − **R**_**29**_(**o**_**hppc**_**s**_**t**_) + **o**_**popgr**_**tx**) − **od**_**nm**_**a**_**t**_

#### Destination health problems (HP) per capita (pc) flows:

Groups need HC pc when their HP pc stock (di_hppc_s immigrants, dn_hppc_s native hosts) top threshold hchppc _ p. They face HC barriers di_hcb_x and dn_hcb_x, in turn, which vary from 0 (none) to 1 (no access). The needed HC for the provision by group (di_tnhcfp_a, dn_tnhcfp_a) and the total HC need for provision (d_tnhcfp_a) take account of the barriers to HC access and the population size by the group. The barrier scenarios by the group can capture varied cases. For example, a case with immigrants having better access to HC in the destination site than in their origin has higher barrier scenario values for the origin site than for the immigrants below.

23. **di**_**tnhcfp**_**a**_**t**_ =  **MAX** (**di**_**hppc**_**s**_**t**_ − **hchppc**_**p**, **0**) ∗ (**1** − **di**_**hcb**_**x**_**t**_) ∗ **di**_**pop**_**s**_**t**_
24. **dn**_**tnhcfp**_**a**_**t**_ =  **MAX** (**dn**_**hppc**_**s**_**t**_ − **hchppc**_**p**, **0**) ∗ (**1** − **dn**_**hcb**_**x**_**t**_) ∗ **dn**_**pop**_**s**_**t**_
25. **d**_**tnhcfp**_**a**_**t**_ = **di**_**tnhcfp**_**a**_**t**_ + **dn**_**tnhcfp**_**a**_**t**_

HC quality by group (di_hcq_a, dn_hcq_a) varies from 0 (futile) to 1 (perfect). It tracks scenarios di_hcq_x and dn_hcq_x, in turn, without conflict and EICC, and it falls when these forces rise above respective damage levels.

26. **di**_**hcq**_**a**_**t**_ = **di**_**hcq**_**x**_**t**_ − **R**_**30**_(**d**_**ci**_**a**_**t**_) − **R**_**31**_(**d**_**eicc**_**x**_**t**_)
27. **dn**_**hcq**_**a**_**t**_ = **dn**_**hcq**_**x**_**t**_ − **R**_**32**_(**d**_**ci**_**a**_**t**_) − **R**_**33**_(**d**_**eicc**_**x**_**t**_)

HC capacity (d_hcc_a) follows scenario d_hcc_x in the absence of EICC and conflict and decline when conflict and EICC exceed damage levels, in turn.

28. **d**_**hcc**_**a**_**t**_ = **d**_**hcc**_**x**_**t**_ − **R**_**34**_(**d**_**ci**_**a**_**t**_) − **R**_**35**_(**d**_**eicc**_**x**_**t**_)

Next, suppose the HC capacity in the destination site suffices for providing the TNFP of HC need in the area (d_tnhcfp_a). In this case, the provided HC per capita by the group (di_phcpc_a, dn_phcpc_a) equals the per capita need for provision.

29. $$ {\displaystyle \begin{array}{l}\mathbf{i}\mathbf{f}\;\mathbf{d}\_\mathbf{tnhcfp}\_{\mathbf{a}}_{\mathbf{t}}\le \mathbf{d}\_\mathbf{hcc}\_{\mathbf{a}}_{\mathbf{t}}:\\ {}\kern0.84em \mathbf{d}\mathbf{i}\_\mathbf{phcpc}\_{\mathbf{a}}_{\mathbf{t}}=\mathbf{di}\_\mathbf{tnhcfp}\_{\mathbf{a}}_{\mathbf{t}}/\mathbf{di}\_\mathbf{pop}\_{\mathbf{s}}_{\mathbf{t}}\\ {}\kern0.84em \mathbf{d}\mathbf{n}\_\mathbf{phcpc}\_{\mathbf{a}}_{\mathbf{t}}=\mathbf{dn}\_\mathbf{tnhcfp}\_{\mathbf{a}}_{\mathbf{t}}/\mathbf{dn}\_\mathbf{pop}\_{\mathbf{s}}_{\mathbf{t}}\end{array}} $$

The model divides HC capacity falling short of the total need for provision (TNFP) of HC in the host area to the groups. In principle, there is more than one way to do it. The model offers two courses (one may add more if so desired). If scenario d_divhcc_x = 1 at t, the model divides the HC capacity by the groups’ shares in the TNFP of HC.

30. **if** **(d_tnhcfp**_**a**_**t**_ > **d_hcc**_**a**_**t**_) **and (d_divhcc**_**x**_**t**_** = 1**): **di_phcpc**_**a**_**t**_** = (di_tnhcfp**_**a**_**t**_**/d_tnhcfp**_**a**_**t**_) **∗ d_hcc**_**a**_**t**_**/di_pop**_**s**_**t**_ **dn_phcpc**_**a**_**t**_ **= (dn_tnhcfp**_**a**_**t**_**/d_tnhcfp**_**a**_**t**_**) ∗ d_hcc** **a**_**t**_**/dn pop**_**s**_**t**_

If d_divhcc_x = 2, groups get scenario shares (di_hccsha_x, dn_hccsha_x) of the capacity.

31. $$ {\displaystyle \begin{array}{l}\mathbf{i}\mathbf{f}\left(\mathbf{d}\_\mathbf{tnhcfp}\_{\mathbf{a}}_{\mathbf{t}}>\mathbf{d}\_\mathbf{hcc}\_{\mathbf{a}}_{\mathbf{t}}\right)\kern0.3em \mathbf{and}\kern0.3em \left(\mathbf{d}\_\mathbf{divhcc}\_{\mathbf{x}}_{\mathbf{t}}=2\right):\\ {}\kern0.96em \mathbf{d}\mathbf{i}\_\mathbf{tphc}\_{\mathbf{a}}_{\mathbf{t}}=\mathbf{di}\_\mathbf{hcc}\mathbf{sha}\_{\mathbf{x}}_{\mathbf{t}}\kern0.4em \ast \kern0.4em \mathbf{d}\_\mathbf{hcc}\_{\mathbf{a}}_{\mathbf{t}}/\mathbf{di}\_\mathbf{pop}\_{\mathbf{s}}_{\mathbf{t}}\\ {}\kern0.96em \mathbf{d}\mathbf{n}\_\mathbf{tphc}\_{\mathbf{a}}_{\mathbf{t}}=\mathbf{dn}\_\mathbf{hcc}\mathbf{sha}\_{\mathbf{x}}_{\mathbf{t}}\kern0.4em \ast \kern0.4em \mathbf{d}\_\mathbf{hcc}\_{\mathbf{a}}_{\mathbf{t}}/\mathbf{dn}\_\mathbf{pop}\_{\mathbf{s}}_{\mathbf{t}}\end{array}} $$

The HP pc flow impacts of the provided HC (di_phchppc_a, dn_phchppc_a) take account of HC quality:

32. **di**_**phchppc**_**a**_**t**_ = **di**_**phcpc**_**a**_**t**_ ∗ **di**_**hcq**_**a**_**t**_
33. **dn**_**phchppc**_**a**_**t**_ = **dn**_**phcpc**_**a**_**t**_ ∗ **dn**_**hcq**_**a**_**t**_

The arrivals’ HP pc (od_imhppc_a) impact on the immigrant HP pc flow (di_imhppc_a) is derived like for the origin. Since people join, it is negative when the arrivals are healthier than immigrants.

34. **di**_**imhppc**_**a**_**t**_ = **od**_**nm**_**a**_**t**_/(**di**_**pop**_**s**_**t**_ + **od**_**nm**_**a**_**t**_) ∗ (**od**_**imhppc**_**a**_**t**_ − **di**_**hppc**_**s**_**t**_)

Natural wear & tear (wthppc_p) raises HP pc and self-healing (shhppc_p) lowers it. EP, EICC, and conflict rising above thresholds, in turn, raise HP. A share of the immigrants’ HP pc (dn_dihppc_p) spills over to affect the natives’ HP pc (including the effect of countermeasures) due to, e.g., infectious diseases and copying habits that impact health (e.g., smoking, diet, exercise). The comparable native spillover share is di_dnhppc_p. TNE impacts follow scenarios di_hppcf_tx and dn_hppcf_tx. HP pc impacts of chance by the group are drawn from a normal distribution with mean 0 and variance vhppc_p, assuming people tend to be healthy. Model variants discerning HP by type or examine cases in which some populations are at high risk of HP (e.g., refugees and asylum-seekers, elderly) would use skewed distributions, as noted.

Next, the model computes the immigrants’ and natives’ HP pc flows (di_hppc_f, dn_hppc_f).

35. **di**_**hppc**_**f**_**t**_ = **wthppc**_**p** − **shhppc**_**p** + **R**_**36**_(**di**_**ep**_**s**_**t**_) + **R**_**37**_(**d**_**eicc**_**x**_**t**_) + **R**_**38**_(**d**_**ci**_**a**_**t**_) + **RD**(**Normal**, **0**, **vhppc**_**p**) + **di**_**dnhppc**_**p** ∗ **dn**_**hppc**_**s**_**t**_ − **di**_**phchppc**_**a**_**t**_ + **di**_**imhppc**_**a**_**t**_ + **di**_**hppcf**_**tx**_**t**_
36. **dn**_**hppc**_**f**_**t**_ = **wthppc**_**p** − **shhppc**_**p** + **R**_**39**_(**dn**_**ep**_**s**_**t**_) + **R**_**40**_(**d**_**eicc**_**x**_**t**_) + **R**_**41**_(**d**_**ci**_**a**_**t**_) + **RD**(**Normal**, **0**, **vhppc**_**p**) + **dn**_**dihppc**_**p** ∗ **di**_**hppc**_**s**_**t**_ − **dn**_**phchppc**_**a**_**t**_ + **dn**_**hppcf**_**tx**_**t**_

#### Destination environmental health problems excluding EICC (EP) flows:

People need ES when the EP stock they face rises above a limit. MAX (di_ep_s - esep_p, 0) gives the ES needed by the immigrants, and MAX (dn_ep_s - esep_p, 0) by the native hosts, where esep_p is the EP threshold for ES need. The groups’ ES barriers (di_esb_x, dn_esb_x) vary from 0 (none) to one (no access). The needed ES for provision by the group (di_tnesfp_a, dn_tnesfp_a) and site (d_tnesfp_a) are given by:

37. **di**_**tnesfp**_**a**_**t**_ =  **MAX** (**di**_**ep**_**s**_**t**_ − **esep**_**p**, **0**) ∗ (**1** − **di**_**esb**_**x**_**t**_) ∗ **di**_**pop**_**s**_**t**_
38. **dn**_**tnesfp**_**a**_**t**_ =  **MAX** (**dn**_**ep**_**s**_**t**_ − **esep**_**p**, **0**) ∗ (**1** − **dn**_**esb**_**x**_**t**_) ∗ **dn**_**pop**_**s**_**t**_
39. **d**_**tnesfp**_**a**_**t**_ = **di**_**tnesfp**_**a**_**t**_ + **dn**_**tnesfp**_**a**_**t**_

Without conflict and EICC in the host area, ES quality by group (di_esq_a, dn_esq_a), which varies from 0 to 1, follows scenario (di_esq_x, dn_esq_x). Otherwise, it falls when conflict and EICC rise above harm levels, in turn. ES capacity (d_esc_a) follows suits with scenario d_esc_x for no EICC and conflict.

40. **di**_**esq**_**a**_**t**_ = **di**_**esq**_**x**_**t**_ − **R**_**42**_(**d**_**ci**_**a**_**t**_) − **R**_**43**_(**d**_**eicc**_**x**_**t**_)
41. **dn**_**esq**_**a**_**t**_ = **dn**_**hcq**_**x**_**t**_ − **R**_**44**_(**d**_**ci**_**a**_**t**_) − **R**_**45**_(**d**_**eicc**_**x**_**t**_)
42. **d**_**esc**_**a**_**t**_ = **d**_**esc**_**x**_**t**_ − **R**_**46**_(**d**_**ci**_**a**_**t**_) − **R**_**47**_(**d**_**eicc**_**x**_**t**_)

If the site’s ES capacity (d_esc_a) suffices for the TNFP of ES (d_tnesfp_a), the ES provision by group (di_tpes_a, dn_tpes_a) equals the group’s needed ES for provision (di_tnesfp_a, dn_tnesfp_a).

43. $$ {\displaystyle \begin{array}{l}\mathbf{i}\mathbf{f}\;\mathbf{d}\_\mathbf{tnesfp}\_{\mathbf{a}}_{\mathbf{t}}\le \mathbf{d}\_\mathbf{esc}\_{\mathbf{a}}_{\mathbf{t}}:\\ {}\kern0.96em \mathbf{d}\mathbf{i}\_\mathbf{tpes}\_{\mathbf{a}}_{\mathbf{t}}=\mathbf{di}\_\mathbf{tnesfp}\_{\mathbf{a}}_{\mathbf{t}}\\ {}\kern0.96em \mathbf{d}\mathbf{n}\_\mathbf{tpes}\_{\mathbf{a}}_{\mathbf{t}}=\mathbf{dn}\_\mathbf{tnesfp}\_{\mathbf{a}}_{\mathbf{t}}\end{array}} $$

If the ES capacity does not suffice for the TNFP of ES, and scenario d_divesc_x = 1 at t, groups get their shares in the overall need out of the ES capacity; if d_divesc_x = 2, the immigrants get scenario share di_escsha_x of the ES capacity, and the native hosts share dn_escsha_x.

44. $$ {\displaystyle \begin{array}{l}\mathbf{if}\;\left(\mathbf{d}\_\mathbf{tnesfp}\_{\mathbf{a}}_{\mathbf{t}}>\mathbf{d}\_\mathbf{esc}\_{\mathbf{a}}_{\mathbf{t}}\right)\kern0.2em \mathbf{and}\kern0.2em \left(\mathbf{d}\_\mathbf{divesc}\_{\mathbf{x}}_{\mathbf{t}}=\mathbf{1}\right):\\ {}\kern0.96em \mathbf{di}\_\mathbf{tpes}\_{\mathbf{a}}_{\mathbf{t}}=\mathbf{di}\_\mathbf{tnesfp}\_{\mathbf{a}}_{\mathbf{t}}/\mathbf{d}\_\mathbf{tnesfp}\_{\mathbf{a}}_{\mathbf{t}}\ast \mathbf{d}\_\mathbf{esc}\_{\mathbf{a}}_{\mathbf{t}}\\ {}\kern0.96em \mathbf{dn}\_\mathbf{tpes}\_{\mathbf{a}}_{\mathbf{t}}=\mathbf{dn}\_\mathbf{tnesfp}\_{\mathbf{a}}_{\mathbf{t}}/\mathbf{d}\_\mathbf{tnesfp}\_{\mathbf{a}}_{\mathbf{t}}\ast \mathbf{d}\_\mathbf{esc}\_{\mathbf{a}}_{\mathbf{t}}\end{array}} $$
45. $$ {\displaystyle \begin{array}{l}\mathbf{if}\;\left(\mathbf{d}\_\mathbf{tnesfp}\_{\mathbf{a}}_{\mathbf{t}}>\mathbf{d}\_\mathbf{esc}\_{\mathbf{a}}_{\mathbf{t}}\right)\kern0.2em \mathbf{and}\kern0.2em \left(\mathbf{d}\_\mathbf{divesc}\_{\mathbf{x}}_{\mathbf{t}}=2\right):\\ {}\kern0.96em \mathbf{di}\_\mathbf{tphc}\_{\mathbf{a}}_{\mathbf{t}}=\mathbf{di}\_\mathbf{esc}\mathbf{sha}\_{\mathbf{x}}_{\mathbf{t}}\ast \mathbf{d}\_\mathbf{esc}\_{\mathbf{a}}_{\mathbf{t}}\\ {}\kern0.96em \mathbf{dn}\_\mathbf{tphc}\_{\mathbf{a}}_{\mathbf{t}}=\mathbf{dn}\_\mathbf{esc}\mathbf{sha}\_{\mathbf{x}}_{\mathbf{t}}\ast \mathbf{d}\_\mathbf{esc}\_{\mathbf{a}}_{\mathbf{t}}\end{array}} $$

The EP flow impacts of the provided ES by group (di_pesep_a, dn_pesep_a) are given by:

46. **di**_**pesep**_**a**_**t**_ = **di**_**tpes**_**a**_**t**_ ∗ **di**_**esq**_**a**_**t**_
47. **dn**_**pesep**_**a**_**t**_ = **dn**_**tpes**_**a**_**t**_ ∗ **di**_**esq**_**a**_**t**_

Individuals create d_ieppc_p EP per period. The total creation is d _ ieppc _ p ∗ (di _ pop _ s_t_ + od _ nm _ a_t_) for the immigrants and d _ ieppc _ p ∗ dn _ pop _ s_t_ for the natives. Share dn_diep_p of the immigrants’ EP spills over to raise the EP facing the natives (e.g., due to wind, dumping). As a result, the immigrants’ EP stock declines. The natives’ spillover share is di_dnep_p. The EP changes are di _ dnep _ p ∗ dn _ ep _ s_t_ − dn _ diep _ p ∗ di _ ep _ s_t_ for the immigrants, and dn _ diep _ p ∗ di _ ep _ s_t_ − di _ dnep _ p ∗ dn _ ep _ s_t_ for the natives. EP stocks decay at the rate d_decrep_p. The EP effects of conflict and EICC track RF functions and the TNE effects scenarios (di_ep_tx, dn_ep_tx).

The next two equations compute the EP flows for the immigrants and natives (di_ep_f, (dn_ep_f):

48. **di**_**ep**_**f**_**t**_ = **d**_**ieppc**_**p** ∗ (**di**_**pop**_**s**_**t**_ + **od**_**nm**_**a**_**t**_) + **di**_**dnep**_**p** ∗ **dn**_**ep**_**s**_**t**_ − **dn**_**diep**_**p** ∗ **di**_**ep**_**s**_**t**_ + **RF**_**21**_(**d**_**ci**_**a**_**t**_) + **RF**_**22**_(**d**_**eicc**_**x**_**t**_) − **di**_**tpesep**_**a**_**t**_ − **d**_**decrep**_**p** ∗ **di**_**ep**_**s**_**t**_ + **di**_**ep**_**tx**_**t**_
49. **dn**_**ep**_**f**_**t**_ = **d**_**ieppc**_**p** ∗ **dn**_**pop**_**s**_**t**_ + **dn**_**diep**_**p** ∗ **di**_**ep**_**s**_**t**_ − **di**_**dnep**_**p** ∗ **dn**_**ep**_**s**_**t**_ + **RF**_**23**_(**d**_**ci**_**a**_**t**_) + **RF**_**24**_(**d**_**eicc**_**x**_**t**_) − **dn**_**tpesep**_**a**_**t**_ − **d**_**decrep**_**p** ∗ **dn**_**ep**_**s**_**t**_ + **dn**_**ep**_**tx**_**t**_

#### Destination population flows:

The immigrants’ natural net birth rate is di_netbr_p and the natives’ dn_netbr_p. Their immigrant population rises due to immigration (od_nm_a), falls when its HP pc stock rises above the death threshold, and depends on a TNE impact. The native group follows suits without arrivals. The population flows (immigrants: di_pop_f, natives: dn_pop_f) are given by:

50. **di**_**pop**_**f**_**t**_ = **di**_**pop**_**s**_**t**_ ∗ (**di**_**netbr**_**p** − **R**_**48**_(**di**_**hppc**_**s**_**t**_) + **di**_**popgr**_**tx**) + **od**_**nm**_**a**_**t**_
51. **dn**_**pop**_**f**_**t**_ = **dn**_**pop**_**s**_**t**_ ∗ (**dn**_**netbr**_**p** − **R**_**49**_(**dn**_**hppc**_**s**_**t**_) + **dn**_**popgr**_**tx**)

#### Stocks and time:

If the end time (endtime_p) has not arrived, the model updates its stock variables using their flows, advances t, and computes another cycle starting in eq. (1); else, it stops.

52. **if t < endtime**_**p:**
  $$ {\displaystyle \begin{array}{l}\mathbf{o}\_\mathbf{pop}\_{\mathbf{s}}_{\mathbf{t}+\mathbf{1}}=\mathbf{o}\_\mathbf{pop}\_{\mathbf{s}}_{\mathbf{t}}+\mathbf{o}\_\mathbf{pop}\_{\mathbf{f}}_{\mathbf{t}}\\ {}\mathbf{o}\_\mathbf{ep}\_{\mathbf{s}}_{\mathbf{t}+\mathbf{1}}=\mathbf{o}\_\mathbf{ep}\_{\mathbf{s}}_{\mathbf{t}}+\mathbf{o}\_\mathbf{ep}\_{\mathbf{f}}_{\mathbf{t}}\\ {}\mathbf{o}\_\mathbf{hppc}\_{\mathbf{s}}_{\mathbf{t}+\mathbf{1}}=\mathbf{o}\_\mathbf{hppc}\_{\mathbf{s}}_{\mathbf{t}}+\mathbf{o}\_\mathbf{hppc}\_{\mathbf{f}}_{\mathbf{t}}\\ {}\mathbf{di}\_\mathbf{pop}\_{\mathbf{s}}_{\mathbf{t}+\mathbf{1}}=\mathbf{di}\_\mathbf{pop}\_{\mathbf{s}}_{\mathbf{t}}+\mathbf{di}\_\mathbf{pop}\_{\mathbf{f}}_{\mathbf{t}}\\ {}\mathbf{di}\_\mathbf{ep}\_{\mathbf{s}}_{\mathbf{t}+\mathbf{1}}=\mathbf{di}\_\mathbf{ep}\_{\mathbf{s}}_{\mathbf{t}}+\mathbf{di}\_\mathbf{ep}\_{\mathbf{f}}_{\mathbf{t}}\\ {}\mathbf{di}\_\mathbf{hppc}\_{\mathbf{s}}_{\mathbf{t}+\mathbf{1}}=\mathbf{di}\_\mathbf{hppc}\_{\mathbf{s}}_{\mathbf{t}}+\mathbf{di}\_\mathbf{hppc}\_{\mathbf{f}}_{\mathbf{t}}\\ {}\mathbf{dn}\_\mathbf{pop}\_{\mathbf{s}}_{\mathbf{t}+\mathbf{1}}=\mathbf{di}\_\mathbf{pop}\_{\mathbf{s}}_{\mathbf{t}}+\mathbf{di}\_\mathbf{pop}\_{\mathbf{f}}_{\mathbf{t}}\\ {}\mathbf{dn}\_\mathbf{ep}\_{\mathbf{s}}_{\mathbf{t}+\mathbf{1}}=\mathbf{dn}\_\mathbf{ep}\_{\mathbf{s}}_{\mathbf{t}}+\mathbf{dn}\_\mathbf{ep}\_{\mathbf{f}}_{\mathbf{t}}\\ {}\mathbf{dn}\_\mathbf{hppc}\_{\mathbf{s}}_{\mathbf{t}+\mathbf{1}}=\mathbf{dn}\_\mathbf{hppc}\_{\mathbf{s}}_{\mathbf{t}}+\mathbf{dn}\_\mathbf{hppc}\_{\mathbf{f}}_{\mathbf{t}}\\ {}\mathbf{t}=\mathbf{t}+\mathbf{1}\\ {}\mathbf{Go}\ \mathbf{t}\mathbf{o}\ \left(\mathbf{1}\right)\mathbf{for}\kern0.5em \mathbf{another}\kern0.5em \mathbf{computation}\kern0.5em \mathbf{cycle}\end{array}} $$
53. **if t** = **endtime**_**p** :  **stop computation**

## Results

We simulate the pilot model for four 60-period host-area storylines (S1-S4) focusing on health problems (HP), health care (HC), population, and sensitivity analyses. The input values are synthetic (as noted), and we compute using Excel. In storyline S1, natives and immigrants have full access to perfect HC and environmental health services (ES). The HC and ES capacities do not change over time. There are no armed conflicts, ecological impacts of climate change (EICC), immigration, and intergroup HP and non-EICC environmental health problems (EP) spillovers. Storyline S2 adds climate migrants and HP spillovers to storyline S1, all else the same. Storylines S3 and S4 add HC barriers and imperfect HC for the immigrants to storyline S2, in turn, keeping all else as in S2.

### Storyline S1: perfect quality without arrival, barriers, & spillovers

Storyline S1 (Table 1) sets the qualities to 1 (perfect), barriers 0 (none), and capacity divisions 1 (short capacity divided by group shares in the need). The HC and ES capacities are 4200. The HC one (we will see) allows shortage; the ES level suffices and, together with our setting of the EP decay and individual creation to 0, focuses ideas on HP. The HP pc impacts of the total non-EICC exogenous (TNE) factors are − 1 by group and t, representing pro-health social determinants of health. Other TNE levels are 0 (to simplify). At t 0, there are 100 immigrants and 2000 natives. Their HP pc and EP stocks are 48 and 47, in turn. The thresholds for needing HC/ES and EP raising HP pc are 48, so at t 0, no one needs HC/ES, and EP does not harm health (stocks ≤ thresholds). The HP pc death level should top the one for HC need. We use 50, so (we will see) death can occur. The net birth rate is 0.1%/period by the group, and HP pc wear & tear is 2/period. The self-healing and the variance of the HP pc impact of chance are 0 (to simplify). The parameters of the rise (R) and rise-fall (RF) functions of conflict, EICC, and EP are 0 (as S1 sets the conflict and EICC levels to zero and, we shall see, generates EP < the threshold for raising HP pc at all t). R_48_(di _ hppc _ s_t_) and R_49_(di _ hppc _ s_t_) for the population flows should give 0 if HP pc ***≤*** death level and rates rising above 0 if the HP pc stock rises above death. One possibility is $\mathrm{R}\left({\mathrm{hppc}}_{\mathrm{t}}\right)=0.001\Big({\mathrm{e}}^{\operatorname{MAX}\left({\mathrm{hppc}}_{\mathrm{t}}-50\right),0\Big)}-1$, by the group.

**Table 1:** Input values for scenario S1

**Controllers**	
Arrival	1 … 1
Conflict	1 … 1
**Scenarios (60 periods)**	
Number of arrivals per period	0 … 0
Arrivals’ HP pc	0 … 0
Conflict intensity	0 … 0
EICC intensity	0 … 0
HC capacity	4200 … 4200
ES capacity	4200 … 4200
Native host HC pc impact of TNE	-1 … -1
Immigrant HC pc impact of TNE	-1 … -1
Native host other TNE impacts	0 … 0
Immigrant TNE impacts	0 … 0
HC quality provided to native hosts	1 … 1
HC barriers facing native hosts	0 … 0
ES quality provided to native hosts	1 … 1
ES barriers facing native hosts	0 … 0
HC quality provided to immigrants	1 … 1
HC barriers facing immigrants	0 … 0
ES quality provided to immigrants	1 … 1
ES barriers facing immigrants	0 … 0
HC/ES capacity splits	1 … 1
**Initial stocks**	
Immigrant population stock	100
Native host population stock	2000
Immigrant HP pc stock	48
Native host HP pc stock	48
Immigrant EP stock	47
Native host EP stock	47
**Parameters**	
End-time	60
Native host HP pc spillover on immigrant HP pc	0
Immigrant spillover on native host HP pc	0
Native EP spillover on immigrant EP pc	0
Immigrant EP spillover on native host EP	0
Native host birth rate	0.001
Immigrant birth rate	0.001
Individually created EP	0
EP decay rate	0
HP pc threshold for HC need	48
EP threshold for ES need	48
EP threshold for impacting HP pc	48
HP pc threshold for death	50
HP pc natural wear & tear	2
HP pc self-healing	0
HP pc variance	0

**Fig. 1:**
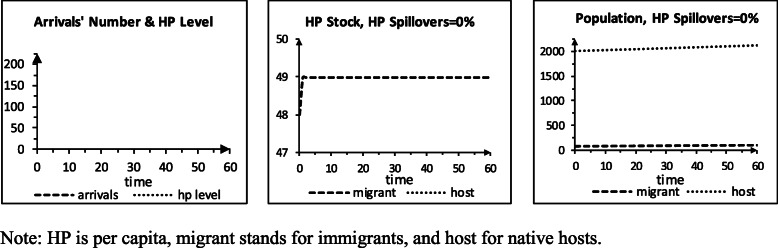
Scenario S1, perfect quality, no arrival, no barriers, no spillovers

Let us walk through the model for storyline S1. At t 0, the groups’ EP and HP pc stocks are below the levels for impacting HP and needing HC, in turn. For the HP pc flows, the effects are 0 for provision, EP, conflict, and arrival; 2 for wear & tear; and −1 for the TNE factors; so, the flows are 1 by the group. The ES needs are 0 (as the associated EP stocks are smaller than the need threshold). As a result, the flow impacts of ES are 0. Other EP flow effects are 0, so the EP flows are 0. The net birth rate is 0.1%. Other population flow impacts are 0. The immigrants’ population flow is 0.1 and native 2. At t 1, the HP pc stocks are 49 [48 (prior) + 1 (flow)], EP stocks 47, the immigrant population 100.1, and the native 2002. The HC needs for provision are 1, by the group [HP pc stock (49) – need threshold (48)]. The groups’ HP pc flows are 0 [2 (wear & tear) – 1 (quality) ∙ 1 (HC) – 1 (TNE)], so the HP pc stocks at t 2 are 49. The EP flows are 0, so the EP stocks are 47. The groups grow at 0.1%/period. This pattern repeats to t 60. Figure 1 shows the results.

### Storyline S2: arrival & HP pc spillovers

Storyline S2 (Table 2) resembles storyline S1, but new immigrants arrive, and health problems spill across the groups. The arrivals’ number/period and HP pc scenarios rise from average levels and then decline to zero, representing the impact of an extreme weather event in the origin site. The decline captures the effects of, e.g., the event’s damages and passing and the destination making it harder to get in (e.g., the United States after Hurricane Mitch). The arrivals’ number is 10 in periods 1–3 and 100 in 4. It rises by 25/period in periods 5–8 and is 0 in 9–60. The arrivals’ HP pc is 48 in periods 1–3 (like the natives’), 55 in periods 4–8, and 0 in 9–60 (as the immigration stops). We set the HP pc spillover shares to 0, 1%, or 2%, in turn, for a sensitivity analysis.

**Table 2:** Input values turning scenario S1 to scenario S2

**Scenarios (60 periods)**	
Number of arrivals scenario (in thousands)	10, 10, 10, 100, 125, 150, 175, 200, 0 … 0
HP pc of arrivals scenario	48, 48, 48, 55, 55, 55, 55, 55, 0 … 0
**Parameters**	
HP pc spillover – native hosts on immigrants, case 1	0
HP pc spillover – immigrants on native hosts, case 1	0
HP pc spillover – native hosts on immigrants, case 2	0.01
HP pc spillover – immigrants on native hosts, case 2	0.01
HP pc spillover – native hosts on immigrants, case 3	0.02
HP pc spillover – immigrants on native hosts, case 3	0.02

**Fig. 2:**
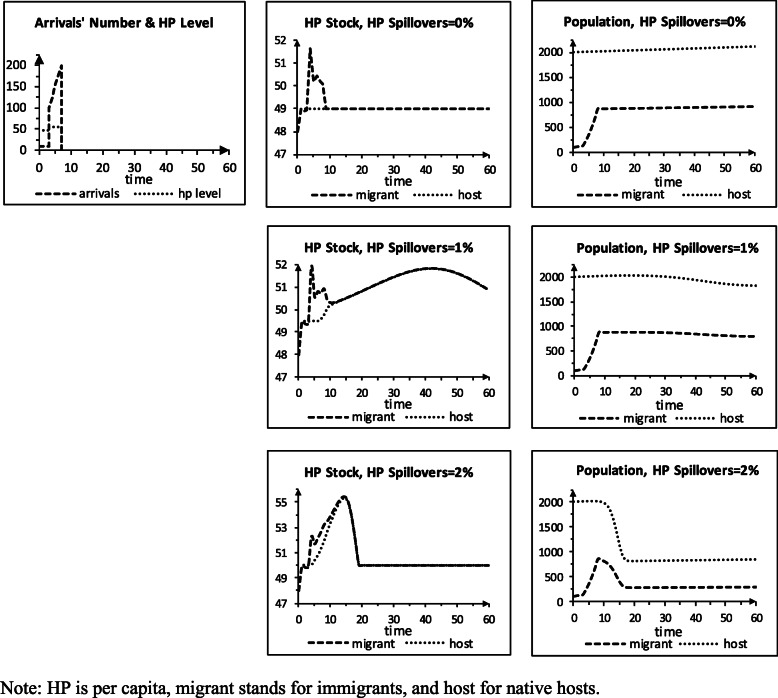
Scenario S2, perfect quality, arrival, no barriers, spillovers

Figure 2 shows the results for storyline S2. For 0% spillovers, the natives’ HP pc is like in S1. At t 1–3, the arrivals are healthier than the resident immigrants, so the group’s HP pc falls. At t 4–8, they are less healthy than their brethren, raising the group’s HP pc and HC need. By t 9, HC provision lowers the immigrants’ HP pc to 49. The native group grows 0.1%/period to t 60. The immigrant group grows at that rate at t 1–3 and less at t 4–8 when its HP pc stock tops the death level (50). By t 9, the groups’ HP pc line up, and the immigrant group again grows 0.1%/period. For 1% spillovers, the immigrants’ HP pc rises higher. The natives’ HP pc increases when the arrivals are less healthy than the immigrants. The total need for provision (TNFP) of HC tops capacity at t 6, and the provided HC falls short of the overall needed level. The HP pc stocks equalize at t 15, peak at t 42, and top death level at t 60. Population decline starts at t 19. The TNFP falls after t 39 as the groups shrink but still tops capacity at t 60. For 2% spillovers, the HP pc stocks exceed the death level even more, and the sizes of the two groups shrink faster. The immigrants have more HP than the natives since they absorb arrivals with HP. The TNFP falls below the HC capacity earlier than for the 1% spillovers, as there are now fewer people. The groups return to grow 0.1%/period but are smaller and less healthy than for the other cases.

### Storylines S3 and S4: arrival, HP spillovers, HC barriers / imperfect quality

**Fig. 3:**
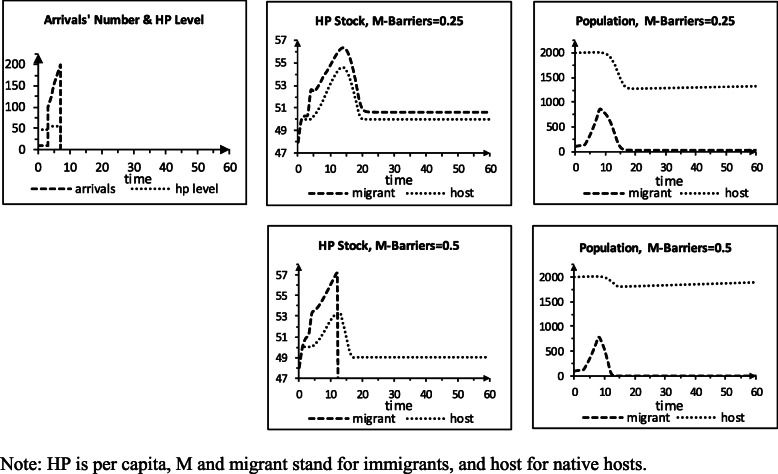
Scenario S3, perfect quality, arrival, barriers for immigrants, 2% spillovers

Storyline S3 (Table 3) resembles storyline S2, except that the immigrant population now faces HC barriers (b) in the host area, and the intergroup HP pc spillovers equal 2%. We examine two b values for sensitivity analysis: immigrants have 75% of the natives’ access (b=0.25) or 50% (0.5). Figure 3 shows the outcomes. For b=0.25, the HC capacity does not suffice to provide the TNFP in periods 2–18. The immigrants now get a smaller share of their HC need than in the 2% spillover & zero barriers to HC simulated in storyline S2. As a result, their HP pc stock rises more, and the population reaches a lower minimum (21 vs. 278) and end value (23 vs. 289). The natives’ HP pc increases less than the immigrants’, as they do not face barriers, and their population has higher minimum (1265 vs. 845) and end (1317 vs. 846) values than in S2. The HP pc stocks steady at 50.01 for the natives and 50.67 immigrants, higher than for the baseline (50). For b=0.5, the immigrants’ HP pc rises more than for b = 0.25, and the immigrant population dies out in period 13. Total HC need then falls below capacity, and the native group again grows 0.1%/period, but it is smaller in period 60 than in period 0 (1898 vs. 2000).

**Table 3:** Input values turning scenario S2 to scenario S3

**Scenarios (60 periods)**	
HC barriers facing immigrants, case 1	0.25 … 0.25
HC barriers facing immigrants, case 2	0.5 … 0.5
**Parameters**	
HP pc spillover – immigrant on native host	0.02
HP pc spillover – native host on immigrant	0.02

**Fig. 4:**
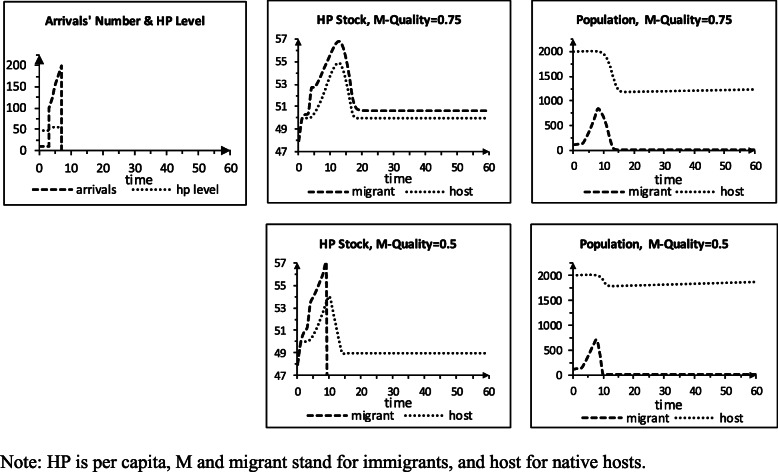
Scenario S4, imperfect quality for immigrants, arrival, no barriers, 2% spillovers

Storyline S4 (Table 4) takes after Storyline S2, but the intergroup HP pc spillovers equal 2%, and the group of the immigrants gets limited HC quality (q) in the host area. We use two q levels for sensitivity analysis: 0.75 (the immigrants get HC with 75% of the natives’ perfect HC level) and 0.5 (they get HC with 50% of the HC quality the natives get). Figure 4 presents the simulation results. For q=0.75, the immigrants’ HP pc stock exceeds the natives’ HP pc stock, and their group population declines. When the immigrants’ HP pc stock stabilizes, they are less healthy than the natives, and their group is nearly gone. For q=0.5, the immigrants’ HP pc stock rises more than it increases for q = 0.75, and their group vanishes. The natives’ HP pc stock then stabilizes at 49, as it did in storyline S1. Their population returns to grow 0.1% per period from a higher level than for q = 0.75 and ends larger at t 60 (1863 vs. 1228).

**Table 4:** Input values turning scenario S2 to scenario S4

**Scenarios (60 periods)**	
HC quality delivered to immigrants, case 1	0.75 … 0.75
HC quality delivered to immigrants, case 2	0.5 … 0.5
**Parameters**	
HP pc spillover – immigrant on native host	0.02
HP pc spillover – native host on immigrant	0.02

### Additional policy comparisons

The policies [b = 0, q = 0.75] in storyline S4 and [b = 0.25, q = 1] in storyline S3 have similar impacts of provided HC on the HP pc flow, for a given HC need. The natives, in turn, get perfect HC quality and face no barriers. With this symmetry, why the HP pc stocks rise sooner and higher for [b = 0, q = 0.75] than for [b = 0.25, q = 1]? To streamline the discussion, we denote the HC capacity c, the HC level the natives need n, and the immigrants’ HC need m. In both cases, the total need for provision (TNFP) > c for a while. If c > TNFP, the provision impact on the HP pc flow is q(1 − b)m for the immigrants and n natives. If c < TNFP, c is divided by the groups’ shares of the TNFP. The immigrants’ share for [b = 0, q = 0.75], $\frac{0.75\mathrm{m}}{\mathrm{m}+\mathrm{n}}$, is smaller than their share for [b = 0.25, q = 1)], $\frac{0.75\mathrm{m}}{0.75\mathrm{m}+\mathrm{n}}$. As a result, they get less HC, and their HP pc climbs sooner and higher for the former policy. This result also holds for the natives ($\frac{\mathrm{n}}{\mathrm{m}+\mathrm{n}}<\frac{\mathrm{n}}{0.75\mathrm{m}+\mathrm{n}}$) and for the policy [b = 0, q = 0.5) versus the policy [b = 0.5, q = 1].

Next, we define the minimum sufficient HC capacity (MSHC) as the smallest level sufficing to provide the TNFP for t 1–60, for each [b, q] policy. Which [b, q] policy has the smallest MSHC? This question has financial implication (capacity building is costly and HC cost rises with its quality) but is hard to answer since higher b raises HP pc and higher q reduces it and both impact the TNFP over time. We can find the MSHC per [b, q] policy by simulating for lower and lower capacity until the capacity first falls below the TNFP. At this point, a slightly larger level will do. In Table 5, policy [b = 0.5, q = 0.5] gives the smallest MSHC and policy [b = 0.25, q = 0.5] the largest. For policy [b = 0.5, q = 0.5], the immigrants’ group vanishes, and the natives’ HP pc stabilizes at 49 by t 24. Policies [b = 0.25, q = 0.5] and [b = 0.5, q = 0.75] stabilize HP pc by t 21 (immigrant 53.4, native 50.1) with end populations (187, 2115). Policy [b = 0.25, q = 0.75] steadies sooner and lower [51.6, 50.03, t = 17] with larger end groups [731, 2119]. Policy [b = 0, q = 1] gives the largest end groups (923, 2123), fastest HP pc steadying (t = 10), lowest steady immigrants’ HP pc (50) and second-lowest natives’ one (50). Which policy is the “best”? DSMs can inform effects of policies but cannot decide for us which is “the best”.

**Table 5:** Minimum sufficient HC capacity

Immigrant HC barriersc	Immigrant HC quality	HC capacity	Immigrant pop t 60	Native pop t 60	Immigrant steady HP	Native steady HP	Time of steady HP
0.25	0.75	7079	731	2119	51.56	50.03	17
0.25	0.5	7758	187	2115	53.38	50.07	21
0.5	0.75	6570	187	2115	53.38	50.07	21
0.5	0.5	6538	0	2119	0	49	24
0	1	6631	923	2123	50	50	10

## Discussion

Are these results credible? The key to evaluating the credibility of dynamic simulation models (DSMs) is the amount of theoretical and empirical evidence supporting their intended use from their development process, performance in simulations, and the quality of the decisions they drive [230, 231]. We apply this evaluation or validation approach to our model.

Our DSM integrates causal processes gleaned from prior empirical results. It is conceptually valid as these processes follow accepted theories and do not merely capture correlations. It is complete for its intended use to the extent that our survey of the prior results is. Our pilot depicts a simplified but not wholly untenable reality. Social science models usually look at composites; ours are simply are more aggregated. Our period of one does not suffice for comparing the model outputs to data but has no algorithmic effect. Modeling one origin-destination (OD) pair is okay if its variables do not depend on other pairs’ variables. Empirical social science models usually make this assumption for their unit of analysis. Modeling total populations is fine, though it prevents studying things by subsets. Our no OD conflict feature usually holds. Relaxing these assumptions is a worthy effort to be discussed. Our design can contain more detail and things we possibly missed; as pilot DSMs go, this is a good thing.

How realistic our input values are? Health care (HC) and environmental health services (ES) with perfect quality and zero barriers, non-EICC environmental health problems (EP) with zero decay and individual creation, and health problems (HP) with zero impacts of chance and self-healing are ideals. Small EP effects on HP and ES (ours are zero) are quite common in the North. Zero climate change impacts in host areas do not exist, but the effects are still relatively small, far enough from the equator and poles. We use these ideal types as a baseline, a method going back to sociologist Max Weber. More HC/ES barriers and lower quality for immigrants than natives are typical; our 25–50% less quality and more hurdles are possible. Fixed HC/ES capacities, and no conflict usually hold for quite long whiles. The total needed HC/ES may exceed the capacity during crises, mainly in the South. An initial 5% immigrant-native ratio and a 0.1%/period net birth rate over a month to several years are in the empirical ballpark. Extreme weather events at times create a rise-fall pattern for emigrants per period and their HP upon landing. Our R function for the population impact of HP pc gives roughly the net birth rate if HP pc rises near the death level; this seems about right, as do HP spillovers like our 0–2%. The total HP pc impact of EP, non-EICC exogenous factors, wear & tear, self-healing, and chance is positive, illustrating that, without perfect HC quality, HP pc must finally top its death level. In sum, our non-ideal input values convey a general sense of realism.

Our simulations generate effects in line with associated theories. Restricting HC quality for a group raises its HP pc stock. HP pc rising above a threshold raises the need for HC, and HP pc rising above a higher threshold raises the death rate. HP spillover from one group to another increases the latter’s HP pc stock. Provided HC with a better quality has a larger healing effect. Unmet HC need raises HP pc. Populations grow at their natural rates when their HP pc is below the threshold for needing HC. The arrival of climate migrants raises the immigrants’ population; arrivals healthier than resident immigrants make the group healthier, and vice versa. HP pc of at least one group rises when the total needed HC tops the HC capacity. The sizes of these effects are imprecise from a real-world view (as our input values are synthetic). Their directions and dynamics are plausible and internally consistent and, like the sizes, react cogently in our sensitivity analyses.

The evidence presented above suggests our DSM suffices for its planned use as a basis for improving model realism. As we turn to this task, it is beneficial to put our work in a general context. Our model suggests societies restricting HC/ES for immigrants may harm all their residents’ health. When the total need for HC tops capacity, communities face a tough choice: whose needs will go unmet? Climate change makes running into this dilemma more likely by increasing HP and conflict risks and immigration speed and size. These points apply anywhere globally, though not necessarily in the same intensity.

As we wait for deep global mitigation of carbon emissions and recalling the large projected migration for this century, our work implies that societies valuing public health may need to adapt. The emerging research on climate migration and health advises easing migration pressure by developing needy origins [18, 20]; this may work, but, with the current tendency of fossil-fuel and beef consumption rise with income, it may step up global warming. Raising HC/ES and entry walls for immigrants may ease capacity stress but harm health for all residents and boost unauthorized entry. Lowering the barriers may require building up HC/ES capacity, crowding out other public projects. With these competing effects, further policy analysis can benefit from more DSM research.

## Conclusion

Recent studies call to develop a dynamic simulation model (DSM) of migration, population, public health, and armed conflict potential under climate change, taking account of other forces. We demonstrated that developing and using such a simulation model can help to understand relationships between these forces and policy implications in different places by changing input values, so it is a worthy endeavor. This paper shows we can start this development by joining system science principles and social science theories and findings and implements a mathematical proof-of-concept for such a model. The prior section motivates using our pilot DSM to identify modeling extensions to make it more realistic and less simplified. This section lays out a path on how to do that.

Separating our pilot’s composite variables by their respective measured subtypes provides a natural starting point for further DSM development. The general forms of the equations in such a DSM would largely resemble ours, though mathematical complexity would rise. For example, a model with two types of health problems, all else as here, will have three more stocks, three more flows, and more equations, functions, auxiliary variables, probability distributions, parameters, scenarios, and initial stock values. Modeling the populations per site by subsets such as age-groups, males, females, immigrant types, immigrant-native families, and immigrants per origin-destination (OD) pair would further complicate things. For example, a model with two origin sites, all else as here, would have six more stocks and six more flows than our pilot. Its algorithm will mostly be like ours but include many more equations, variables, and parameters. Other extensions to increase realism include adding OD conflict (using our conflict algorithm), conflict proneness per site (using skewed distributions), stochastic extreme weather events per site (using our conflict method), and delays in the realization of effects.

With a more realistic DSM of climate migration and population health defined, the next stage of the model development is to set a real world-oriented period size and compare the computed outputs to their associated observed data. Finding a suitable period size may require iterative simulations, as a shorter period increases resolution but can create artificial instabilities. Scenarios for the exogenous variables would come from published sources. The model’s parameters and function values could come from expert opinions, reported empirical results, and calibration (i.e., adjusting these values to improve the model fit to data).

The calibration effort, in turn, may proceed visually with graphs and tables or computationally by minimizing a certain fit function (e.g., $\mathrm{FIT}=\sum \limits_{\mathrm{t}=0}^T{\left({\mathrm{y}}_{\mathrm{t}}-{\mathrm{y}\mathrm{d}}_{\mathrm{t}}\right)}^2$ – where y_t_ is output at time t, yd_t_ the related data point, and T the number of periods – by choosing parameters and function values within prespecified ranges around their empirical estimates. This effort may use all the available data for the exogenous variables, ensuring the simulation’s outputs make sense, or use part of the data and compare the results to the portion set aside.

Generating meaningful projections of climate migration and population health by the storyline requires a DSM whose outputs sufficiently match data. Comparing forecasts between existing DSMs may provide further insight, though we think that this option is currently not available in our case. One may also validate DSMs by applying policies and comparing their actual effects to their forecasts. Taking this approach here should proceed with care, as things not working as projected may harm people.

Indeed, projections of fully validated DSMs may not emerge precisely even if the past policies continue, as no one knows the inputs values for the projected horizon. Scientifically assessing the future climate migration and public health trajectories for any given storyline is the unavoidably heaping projection of the variables of direct interest upon forecasts of other variables, raising the potential for a difference between the actual and the model’s projected trajectories. DSMs might require revision now and then, even if their projection is deemed close enough to reality in some cases. New data coming on board may prompt modification of parameters, scenarios, functions, and even equations. In a sense, the process of developing and validating DSMs for conditional policy advice never really comes to an end.

## Supplementary information

**Additional file 1: Appendix.** Defining the model variables and parameters by type and alphabetical.

## Data Availability

Not applicable.

## Abbreviations

DSM: Dynamic simulation model
EICC: Ecological impacts of climate change
EP: Environmental health problems other than EICC (or non-EICC environmental health problems)
ES: Environmental health services
HC: Health care
HIV/AIDS: Human immunodeficiency virus/acquired immunodeficiency syndrome
HP: Health problems
MSHC: Minimum sufficient HC capacity
OD: Origin-destination
pc: Per capita
PIMs: Authorized permanent international immigrants
TIMs: Authorized temporary international immigrant
TNE: Total non-EICC exogenous
TNFP: Total need for provision

## Footnotes

1 We define the abbreviations when introduced, at the end, and, as a reminder, in a few other places.

2 The departing HP is od _ emhppc _ a_t_ ∗ od _ nm _ a_t_. The origin’s total HP is o _ hppc _ s_t_ ∗ o _ pop _ s_t_. Ceteris paribus, the origin’s HP pc stock in t + 1 is o _ hppc _ s_t + 1_ = (o _ hppc _ s_t_ ∗ o _ pop _ s_t_ − od _ emhppc _ a_t_ ∗ od _ nm _ a_t_)/(o _ pop _ s_t_ − od _ nm _ a_t_). Subtracting o _ hppc _ s_t_ from both sides gives (14).

3 For example, hemoglobin, body temperature [226], blood pressure, pulse rate, BMI, diabetes diagnosis age [227], total cholesterol [224], iron, glucose [225], are typically roughly normally distributed. Prostate PSA [228], homocysteine, paranoia [229], cancer clusters [224], and triglycerides [225] are not.
