# Supplementary material for: Climate-related migration and population health: social science-oriented dynamic simulation model
Source: BMC Public Health. 2021 Mar 26;21:598. doi: 10.1186/s12889-020-10120-w (PMC7996123; doi:10.1186/s12889-020-10120-w)
Supplement: Supplementary file 1 — Additional file 1: Appendix. Defining the model variables and parameters by type and alphabetical. [file 12889_2020_10120_MOESM1_ESM.docx]

**Appendix: Defining the model variables and parameters by type and alphabetically**

**Stocks**

di_ep_s EP stock facing the immigrant population in the destination

di_hppc_s HP pc stock of the immigrant population in the destination

di_pop_s immigrant population stock in the destination

dn_ep_s EP stock facing the native host population in the destination

dn_hppc_s HP pc stock of the native host population in the destination

dn_pop_s native host population stock in the destination

o_ep_s EP stock facing the population in the origin

o_hppc_s HP pc stock of the population in the origin

o_pop_s population stock in the origin

**Flows**

di_ep_f EP flow facing the immigrant population in the destination

di_hppc_f HP pc flow of the immigrant population in the destination

di_pop_f immigrant population flow in the destination

dn_ep_f EP flow facing the native host population in the destination

dn_hppc_f HP pc flow of the native host population in the destination

dn_pop_f native host population flow in the destination

o_ep_f EP flow facing the population in the origin

o_hppc_f HP pc flow of the population in the origin

o_pop_f population flow in the origin

**Auxiliaries**

d_ci_a conflict intensity in the destination

d_cr_a conflict risk in the destination

d_esc_a ES capacity in the destination

d_hcc_a HC capacity in the destination

d_nm_a destination effect on the number of immigrants per period

d_tnesfp_a total ES need for provision in the destination

d_tnhcfp_a total HC need for provision in the destination

di_esq_a ES quality provided to immigrants in the destination

di_hcq_a HC quality provided to immigrants in the destination

di_imhppc_a impact of the arrivals’ HP pc on the immigrant HP pc flow in the destination

di_pesep_a impact of ES provided to the immigrant group in the destination on the EP it faces

di_phcpc_a provided HC per capita for the immigrant group in the destination

di_phchppc_a impact of HC provided to immigrants in the destination on their HP pc flow

di_tnesfp_a immigrants’ total needed ES for provision in the destination

di_tnhcfp_a immigrants’ total needed HC for provision in the destination

di_tpes_a total provided ES to the immigrant group in the destination

dn_esq_a HC quality provided to immigrants in the destination

dn_hcq_a HC quality provided to native hosts in the destination

dn_pesep_a impact of ES provided to the native host group in the destination on the EP it faces

dn_phcpc_a provided HC per capita for the native host group in the destination

dn_phchppc_a impact of HC provided to native hosts in the destination on their HP pc flow

dn_tnesfp_a native hosts’ total needed ES for provision in the destination

dn_tnhcfp_a native hosts’ total needed HC for provision in the destination

dn_tpes_a total provided ES to the native host group in the destination

o_ci_a conflict intensity in the origin

o_cr_a conflict risk in the origin

o_emhppc_a origin’s HP pc flow impact of emigration in the origin

o_esc_a ES capacity in the origin

o_esq_a ES quality provided to people in the origin

o_hcc_a HC capacity in the origin

o_hcq_a HC quality provided to people in the origin

o_nm_a origin effect on the number of immigrants per period

o_phchppc_a origin’s HP pc flow impact of provided HC in the origin

o_tnesfp_a total needed ES for provision in the origin

o_tnhcfp_a total needed HC for provision in the origin

o_tpesep_a origin’s EP flow impact of provided ES in the origin

od_imhppc_a HP pc of immigrants from the origin upon arrival to the destination

od_nm_a the number of immigrants from the origin to the destination per period

**Scenarios**

d_ci_tx destination’s conflict intensity impact of the destination’s TNE factor

d_cr_tx destination’s conflict risk impact of the destination’s TNE factor

d_divesc_x ES capacity division way in the destination

d_divhcc_x HC capacity division way in the destination

d_eicc_x EICC composite in the destination

d_hcc_x HC capacity in the destination in the absence of conflict and EICC

d_nm_tx migrants number impact of destination’s TNE factor

di_esb_x ES barriers facing the immigrant group in the destination

di_escsha_x ES capacity share provided to the immigrants in the destination

di_esq_x ES quality provided to immigrants in the destination in the absence of conflict and EICC

di_hcb_x HC barriers facing the immigrant group in the destination

di_escsha_x ES capacity share provided to the immigrants in the destination

di_hccsha_x HC capacity share provided to the immigrants in the destination

di_hcq_x HC quality provided to immigrants in the destination in the absence of conflict and EICC

di_hppcf_tx immigrants’ HP pc impact of the destination’s TNE factor

di_popgr_tx destination immigrant population growth rate impact of the group’s TNE factor

dn_esb_x ES barriers facing the native host group in the destination

dn_escsha_x ES capacity share provided to the native hosts in the destination

dn_esq_x ES quality provided to native hosts in the destination in the absence of conflict and EICC

dn_hcb_x HC barriers facing the native host group in the destination

dn_hccsha_x HC capacity share provided to the native hosts in the destination

dn_hcq_x HC quality provided to native hosts in the destination in the absence of conflict and EICC

dn_hppcf_tx native hosts’ HP pc impact of the destination’s TNE factor

dn_popgr_tx native population growth rate impact of the group’s TNE factor

o_ci_tx origin’s conflict intensity impact of the origin’s TNE factor

o_cr_tx origin’s conflict risk impact of the origin’s TNE factor

o_eicc_x EICC composite in the origin

o_esb_x ES barriers facing people in the origin

o_esc_x ES capacity in the origin site in the absence of conflict and EICC

o_esq_x ES quality delivered in the origin site in the absence of conflict and EICC

o_hcb_x HC barriers facing people in the origin

o_hcc_x HC capacity in the origin site in the absence of conflict and EICC

o_hcq_x HC quality delivered to people in the origin site in the absence of conflict and EICC

o_hppc_tx origin’s HP pc flow impact of the origin’s TNE factor

o_nm_tx migrants number impact of the origin’s TNE factor

o_popgr_tx impact of origin population TNE factor on its growth rate

od_nmhppc_x emigrant to origin HP pc ratio (value > 1 means less healthy)

**Parameters**

d_ieppc_p individually created EP per period in the destination

di_dnep_p share of EP facing native hosts spilling over to impact the EP facing immigrants

di_dnhppc_p share of native hosts’ HP pc spilling over to impact the immigrants’ HP pc

di_netbr_p natural net birth rate (birth rate – death rate) of the immigrant group in the destination

dn_diep_p share of EP facing immigrants spilling over to impact the EP facing native hosts

dn_dihppc_p share of immigrants’ HP pc spilling over to impact the native hosts’ HP pc

dn_netbr_p natural net birth rate (birth rate – death rate) of the native host group in the destination

esep_p EP stock threshold above which people need ES

hchppc_p HP pc stock threshold above which people need HC

o_decrep_p EP stock natural rate of decay in the origin

o_ieppc_p individually created EP per period in the origin

o_netbr_p natural net birth rate (birth rate – death rate) of the population in the origin

shhppc_p HP pc flow impact of self-healing

vhppc_p variance of the HP pc flow impact of chance

wthppc_p HP pc flow impact of natural wear & tear
